# Association between untreated carious lesions and asthma in adults at Rabat University Hospital, Morocco: a cross sectional study

**DOI:** 10.1186/s13104-017-2548-2

**Published:** 2017-06-26

**Authors:** Sanaa Chala, Saloua Rouiffi, Mouna Soualhi, Jamal Eddine Bourkadi, Redouane Abouqal, Faïza Abdallaoui

**Affiliations:** 1 Research Team on Oral Ecosystem, Department of Endodontic and Restorative Dentistry, Faculty of Dentistry, Mohammed V University in Rabat, Rabat, Morocco; 2 Laboratory of Biostatistics, Clinical and Epidemiological Research (LBRCE). Faculty of Medicine and Pharmacy, Mohammed V University in Rabat, Rabat, Morocco; 3 Mohammed V Military Teaching Hospital, Rabat, Morocco; 4 Faculty of Dental Medicine, Mohammed V University in Rabat, Rabat, Morocco; 5 Department of Respiratory Diseases, Moulay Youssef Hospital, Rabat, Morocco Faculty of Medicine and Pharmacy, Mohammed V University in Rabat, Rabat, Morocco; 6 Faculté de Medecine Dentaire de Rabat, BP: 6212. Rabat Instituts, Rabat, Morocco

**Keywords:** Asthma, Dental caries, Social determinants, Oral health behaviors

## Abstract

**Background:**

Depending on risk factors, cumulative risk of developing more dental caries by age has been reported. However, the association between dental caries and asthma is less studied among adults. The aims of this study were to evaluate the severity of untreated carious lesions in a population of asthmatic adults and to evaluate the mediation of socio-economic and oral health behaviour variables.

**Methods:**

The study involved participants with diagnosed asthma (N = 100) and control (N = 100) subjects attending a tertiary hospital. Groups were matched by age and gender. Asthma was the exposure of interest. The outcome measure was the rate of carious lesions. Recorded variables included demographics, socio-economic status, tooth brushing habits and oral health status using WHO criteria. Poisson regression analysis examined the association between asthma and untreated dental caries.

**Results:**

The adjusted model, after the inclusion of individuals’ behaviours regarding oral health, social determinants and asthma, revealed a significant association between the number of untreated carious lesions and asthma (PR = 1.23; 95% CI 1.23–1.58; *p* < 0.001).

**Conclusion:**

Patients with asthma showed a greater number of untreated carious lesions. Looking forward, better understanding of the association between asthma and oral health may require exploiting the interactions of behavioural, social determinant and biological factors.

## Background

Asthma is a common chronic condition that was still rising in prevalence in many countries during the last decades of the twentieth century but has stabilized in others [1, 2].

Like asthma, dental caries is a common public health condition particularly prevalent among high-risk individuals [3].

These two conditions result in both a burden on patient quality of life as well as tremendous socio-economic impact [4, 5].

Over decades many papers have studied the association between asthma and oral health. Accumulating evidence suggests that asthmatic patients have an increased risk of dental caries [6, 7]. However, the pattern of association of asthma and dental caries has been more frequently studied among children.

Because adults normally are more prone to dental caries due to cumulative effects as they age, we conducted a retrospective cohort study of untreated dental caries severity among asthmatic adults. We hypothesized that those adults who have asthma, as compared with adults without asthma, would have an increased risk of having more tooth decay. Furthermore, we evaluated the mediation of socio-economic factors and oral health behaviours.

## Methods

To test the hypothesis that asthma is associated with high numbers of untreated carious lesions, cross-sectional analyses were performed.

Between October 2013 and May 2014, asthmatic patients aged 18 years or over attending Rabat University Hospital’s Department of Allergy and Pulmonary Diseases were invited to participate in the study. Controls were patients treated at the Rabat Dental School during the same period. Informed consent was gained from the participants before inclusion. The study protocol was performed in accordance with ethics principles.

Criteria for inclusion were formulated to exclude adults treated for any other chronic diseases condition (e.g. cardio-vascular diseases, diabetes, etc.) or seeking the dental hospital for dental emergency needs. Cases and controls were matched by age and gender.

### Data collection

Data were gathered through interview and oral examination.

Characteristics of the study population included:

- Demographic and socio-economic variables: age, gender, monthly household income (low, medium or high) and educational level (none, primary, secondary or university);
- Asthma status: yes or no;
- Oral health behaviours: information on frequency of tooth brushing (never, ≤2 per day, >2 per day), regularity, age at beginning tooth brushing, the duration of tooth brushing and the brushing motion (appropriate or inappropriate); and,
- The nature of previous dental attendance, noted according to two categories: acute visit, aesthetic concern.

The intra-oral examinations, based on 28 teeth, used WHO criteria and included the decayed, missing and filled teeth (DMFT) index. Caries was recorded with tooth as the unit of measurement. Examinations were conducted using portable lamps and sterilized instruments.

Gingival health status was determined using the Löe-Silness gingival index (GI), and plaque status using Silness-Löe plaque index (PI). The mean score for each patient was used to classify patients in regression analysis as follows:

- The GI was categorized: mild inflammation (0.1–1.0); moderate inflammation (1.1–1.9); and severe inflammation (2–3).
- The PI was categorized: good (0.1–1.0), fair (1.0–1.9) and poor (2–3).

Interrater reliability for carious lesions recording was performed on 20 participants using Cohen’s kappa statistic. Interrater reliability of the carious lesion recording was 0.89.

### Statistical analysis

Data analysis was conducted using SPSS 13.0 software (Chicago, IL, USA). The data were presented as the mean ± standard deviation (SD) for continuous variable with a normal distribution, and as median with inter-quartile range (IQR) for variables with skewed distribution. For categorical variables, data were presented as proportion.

Comparisons between categorical variables were determined using the Chi square test. Normally distributed continuous variables were analysed by Student’s *t* test. Comparisons between abnormally distributed variables were done by Mann–Whitney *U* test.

We modelled the probability of having more carious lesions using Poisson regression analysis. Unadjusted and adjusted prevalence ratios (PR) and related 95% confidence intervals of untreated carious lesions were calculated. Explanatory variables to be included on multivariable analysis were first identified in univariate analysis. The adjusted prevalence ratios were considered statistically significant when *p* values were 0.05 or less.

## Results

Two hundred adults were included during the study periods, of whom half were control. The mean age of participants was 36.3 ± 15.1 years and 152 (76%) were female. Fifty-one (25.5%) participants had no formal education.

Twenty-two (22%) of the asthma patients had severe asthma. The median exposure time to asthma drugs was 9 (5–17) years. Oral corticosteroid (OCS) combined with inhaled salbutamol and inhaled corticosteroids were used by 43% of asthmatics. Twenty-three (23%) of the asthmatic patients inhaled their asthma medication more than twice a day in all seasons.

The DMFT score of all patients was 16.1 ± 7.6. The prevalence of untreated carious lesions in the study population was 44.03%. The median PI score for study subjects was 1.5 (1–2) and the median GI score was 1.25 (0.64–2).

Characteristics of patients according to asthma are shown in Table 1.

**Table 1:** Demographic, socio-economic, oral health and clinical characteristics of studied groups according to their asthmatic status

	Asthma		p
	Yes (N = 100)	No (N = 100)	
Age (years), (mean ± SD)	36.4 ± 15.2	36.2 ± 15.1	0.94
Female gender, n (%)	76 (76)	76 (76)	>0.99
Education level, n (%)			0.24
Non	31 (31)	20 (20)	
Primary	18 (18)	18 (18)	
Secondary	23 (23)	33 (33)	
University	28 (28)	29 (29)	
Income, n(%)			0.13
Low	39 (39)	28 (28)	
Medium	34 (34)	47 (47)	
High	27 (27)	25 (25)	
Age at beginning tooth brushing (years), [median (IQR)]	12 (7–20)	10 (7–19)	0.54
Duration of brushing, (min), [median (IQR)]	2 (1–3)	2 (1–3)	0.41
Inappropriate brushing motion, n(%)	48 (48)	51 (51)	0.61
DMFT, (mean ± SD)	18.4 ± 8.1	13.8 ± 6.4	<0.001
DT, (mean ± SD)	8.8 ± 4.6	6.2 ± 4.3	<0.001
MT, [median (IQR)]	1 (0–3)	0 (0–1)	<0.001
Proximal DT, [median (IQR)]	4 (1–7)	1 (0–3)	<0.001
Number of WSL at cervical mergins, [median (IQR)]	0 (0–4)	0 (0–1)	<0.001
PI, [median (IQR)]	1.2 (0.8–2)	1.2 (0.5–1.7)	<0.001
GI, [median (IQR)]	1.6 (1–2.6)	1.3 (0.9–1.9)	0.07

IQR, interquartile range; SD, standard deviation; MT, number of missed teeth; DT, number of decayed untreated teeth; DMFT, number of decayed, missed and filled teeth; GI, gingival index; PI, plaque index; WSL, white spot lesions; min, minutes; Proximal DT, number of proximal decayed untreated teeth

No significant difference between asthmatic and control groups was found with regard to oral hygiene practices.

Untreated carious lesions consisted of 48.09% of the DMFT among the asthmatic group.

The distribution of carious lesions profile according to tooth type was statistically different between asthma and non-asthma patients.

Results of Poisson regression analysis are shown in Table 2.

**Table 2:** Uni and multi-variate analysis of factors associated with the number of untreated carious lesions

	DT (m ± SD)	Univariate analysis			Multivariate analysis		
		PR	95% CI	p	PR	95% CI	p
Age, years		1.002	0.99–1.005	0.36			
Female gender	7.5 ± 4.2	1.03	0.91–1.16	0.6			
Occupational status							
Without	8 ± 3.9	1.86	1.07–3.22	0.02	1.97	1.07–3.60	0.05
Student	6.2 ± 4.3	1			1		
Public employment	6.7 ± 5.3	1.6	0.91–2.81	0.09	2.00	1.09–3.66	0.02
Private employment	10.1 ± 6	2.33	1.33–4.08	0.003	2.68	1.43–5	0.002
Education level							
No	8.2 ± 4.8	1.22	1.08–1.37	0.002	1.08	0.88–1.32	0.42
Primary	8.8 ± 4.7	1.34	1.15–1.49	<0.001	1.13	0.93–1.36	0.21
Secondary and university	6.7 ± 4.5	1			1		
Income							
Low	9 ± 4.3	1.42	1.24–1.63	<0.001	1.12	0.86–1.46	0.38
Medium	7.1 ± 4.4	1.12	0.98–1.28	0.08	1.30	1.03–1.65	0.02
High	6.3 ± 4.8	1			1		
Nature of previous dental attendance							
Acute visit	9.2 ± 4.8	1.28	1.07–1.53	0.005	1.37	1.09–1.72	0.006
Aesthetic concern	7.2 ± 4.1	1			1		
Frequency of teeth bruising							
Never	7.9 ± 4.6	1.24	1.06–1.45	0.006	1.26	1.08–1.47	0.003
≤2 per day	7.7 ± 4.7	1.17	1.008–1.37	0.03	1.07	0.85–1.85	0.53
>2 per day	6.3 ± 4.3	1			1		
Duration of teeth brushing		0.98	0.94–1.01	0.32			
Age at beginning teeth brushing		1.01	1.005–1.01	<0.001	1.001	0.99–1.009	0.84
Plaque index		1.28	1.20–1.36	<0.001	1.13	0.92–1.38	0.22
Good	5.6 ± 4.4	1			1		
Fair	7.1 ± 4.5	1.2	1.08–1.4	0.002	1.17	0.95–1.6	0.10
Poor	9 ± 4.5	1.5	1.3–1.8	<0.001	1.23	0.96–1.42	0.11
Gingival index							
Mild	6.4 ± 4.4	1			1		
Moderate	7.4 ± 4.8	1.16	1.19–1.55	<0.001	0.87	0.70–1.09	0.24
Sever	8.7 ± 4.2	1.3	1.02–1.3	0.01	1.04	0.88–1.24	0.59
Asthma							
Yes	8.8 ± 4.6	1.42	1.28–1.57	<0.001	1.39	1.23–1.58	<0.001
No	6.2 ± 4.3	1			1		

DT, number of decayed untreated teeth; 1, reference category; PR, prevalence ratio; CI, confidence interval; m ± SD, mean ± standard deviation

Univariate regression analysis showed that occupational status, low and medium education level, low and medium income, emergency dental attendance, never and 1 to 2 per-day frequency of tooth brushing, fair and poor plaque index, moderate and severe gingival inflammation, and asthma status were all identified as risk factors associated with a higher rate of dental caries.

Adjustment for these factors showed that occupational status, emergency dental attendance, frequency of tooth brushing and asthma status were associated with high rate of dental caries.

## Discussion

The results of this analysis showed that the number of untreated carious lesions were higher among the asthma group. This result was observed in both univariate and multivariate analyses.

The present results are in line with those reported by Anjomshoaa et al. [8] and Johnston L, Vieira AR (2014). Both studies suggested the existence of an association between higher caries experience and asthma in adults [8, 9].

Yet there is little information available to document whether adults with asthma have worse oral health indicators than those without asthma. Most published studies were performed in children [10, 11] and in some cases conflicting results were found. Inconsistent results may be explained by differences in studied populations. Another explanation may be the chronic nature of dental caries. This explanation means that a period of time is required for the risk factors to contribute to the development of this disease. The exposure time to environmental factors that contribute to dental caries development in younger subjects may be shorter than that in adults. As such, the longer exposure time to asthma medications and behavioural factors means that the contribution of asthma and asthmatic medications to dental carious lesion development could be masked by the influence of other probable common risk factors.

The present study explored the association between asthma and the severity of untreated carious lesions. It is worth pointing out that most of previous studies used the DMFT index as indicator of dental caries; a claim-based definition of indicators in this study was to assess and compare untreated carious lesions that may have resulted in healthcare resource use [12–14].

Dental caries is a multi-factorial chronic disease. It cannot develop without interaction with intermediary and environmental factors [15]. Factors like socio-economic background, oral health behaviours and eating habits may interact to explain the disease’s prevalence. Socio-economic differences in oral health status have been reported in many studies [16, 17]. It is important to note that both studied groups have low educational background and social level. Another important specification of the studied group is that a large proportion of adults have unfavourable oral health behaviours. Socio-economic status and oral health behaviours may confer additional disadvantages on carious lesion severity at tooth level [18]. These factors are strongly linked to dental caries and may mediate the link between asthma [19] and dental caries severity.

The mechanism of how asthma is associated with dental caries is still under exploration. Potential associations between asthma and the dental caries process cannot be solely attributed to one specific factor, but to a complex interaction among many factors including genetic factors, environmental exposures, nutrition, socio-economic status and access to health care. Asthmatic patients exposed to the same levels of environmental risk factors may not have the same susceptibility or resistance to dental caries.

A possible linkage between asthma and the dental caries process is genetic factors. Candidate gene categories to date include beta-defensin 1 [20–23] and matrix metalloproteinases [24, 25].

Asthma involves the immune system, and may disturb oral microbiota. Combined with anti-asthma medications [26, 27] this condition, may increase the risk of dental caries development at the individual level. As result, dysbiosis might increase the susceptibility to dental caries in asthmatic patients [26, 27].

Finally, depending on oral health behaviour, social status, genetic factors, and oral environment, the cumulative risk of developing more carious lesions may be more higher among asthmatic patients with genetic predisposition.

While several factors are associated with high risk of dental caries, exposure to some risk factors (i.e., limitations in oral health literacy, misconception about preventive measures, impairments in cognition) could be prevented.

Thus, community-based oral disease prevention and health promotion programs targeted to asthma patients should look beyond medications to cover differences in oral health behaviours, social factors, and other predisposing factors.

Asthma patients with high numbers of untreated carious lesions may require higher total health care costs and consequently a substantial economic cost to society.

Based on their current caries status or markers of disease, a high-risk strategy aiming to target asthma patients should be adopted. This simple public health intervention may lead to a significant reduction of the burden of dental caries and to its cost effectiveness among asthma adults.

The present study has some limitations that should be taken into account. The present findings involved a restricted group of patients and may underestimate the real burden among patients with limited resources and no access to care.

The socio-economic status was quite different between asthma and control groups. Thus, we caution that the present results may not reflect the mediation of socio-economic status in carious lesion severity. Further research design taking into account matching asthma and control groups according to demographic and socio-economic background is needed.

Despite these limitations, the current report provides useful information about the factors that should be analysed when comparing groups regarding their exposure factors.

## Conclusions

In cross-sectional analysis of present data, the presence of asthma was associated with a greater number of untreated carious lesions. Further studies are warranted to explore the role of common risk indicators.

## Abbreviations

PR: prevalence ratios
SD: standard deviation
CI: confidence interval
DT: decayed untreated teeth
DMFT: decayed missed and treated teeth
